# Perceived barriers of access to health and nutrition services under drought and food insecurity emergency in north-east Uganda: a qualitative study

**DOI:** 10.1186/s12889-024-17830-5

**Published:** 2024-02-06

**Authors:** Charles Njuguna, Habteyes Hailu Tola, Benson Ngugi Maina, Kwikiriza Nicholas Magambo, Nabunya Phoebe, Elizabeth Adhiambo Mgamb, Evelyne Tibananuka, Florence M. Turyashemererwa, Moses Rubangakene, Kisubika Richard, George Opong, Ssekitoleko Richard, Chris Opesen, Tim Mateeba, Edmond Muyingo, Upenytho George, Samalie Namukose, Yonas Tegegn Woldemariam

**Affiliations:** 1 World Health Organization Uganda Country Office, Plot 60 Prince Charles Drive, Kampala, Kololo, P. O. Box: 24578, Uganda; 2 Ministry of Health of Uganda, Kampala, Uganda

**Keywords:** Access to health services, Health services delivery, Nutrition service, Health system service, Drought, Food insecurity

## Abstract

**Background:**

In the face of drought and food insecurity emergency, evidence on access to health and nutrition services is important. Karamoja is one of the regions that have experienced extreme drought and food insecurity emergency in Uganda. As a part of the drought and food insecurity emergency response, World Health Organization (WHO) with Ministry of Health (MoH) has designed and implemented a qualitative study in 15 districts that have experienced drought and food insecurity emergency in north-east Uganda. Thus, we aimed to explore the barriers of access to health and nutrition services in drought and food insecurity emergency affected districts in north-east Uganda.

**Methods:**

We employed a descriptive qualitative study design. We interviewed 30 patients and 20 Village Health Teams (VHT) from 15 districts. We employed an in-depth interview with semi-structured questions to collect data until information saturation reached. We used thematic data analysis approach by ATLAS.ti version 7.5.1.8 software.

**Results:**

Of the 30 interviewed subjects, 15 were female, and the median age of the subjects was 29 years with interquartile range (IQR) of 23 to 37 years. Majority (68.8%) of subjects reported that access to health and nutrition services was harder to them. Four themes: sociocultural and economic; environmental; health system, and individual related factors were identified as the barriers of access to health and nutrition services.

**Conclusion:**

The present study identified several modifiable barriers that hinder access to health and nutrition services in drought and food insecurity affected districts. Comprehensive interventions aimed at addressing sociocultural, economic, environmental, health system and subject related challenges are required to improve access to health and nutrition services in drought and food insecurity affected setups.

## Background

Access to quality healthcare services is one of the key health related sustainable development goals (SDGs) targets [1]. It is also becoming an important point of discussion across the world due to its impact on health intervention outcomes, and the value it has to achieve other SDGs targets. However, access to health services continues to be a challenge across the world [2]. A recent estimate shows that half of the world population do not have access to essential health services, and 100 million people are still strapped into extreme poverty because of health-related expenses [2]. Particularly, population who are living in low- and middle-income countries (LMICs) have limited access to quality healthcare services [3, 4]. For instance, more than half (52%) of the African population have no access to essential healthcare services [3], which could contribute to high burden of preventable and treatable diseases. Existing evidence indicates that considerable proportion of morbidity and mortality due to preventable and treatable diseases are attributed to inadequate access to quality healthcare services [5]. For instance, of 8.6 million excess deaths occurred due to healthcare system related problems in 61 LMICs, five million of them occurred due to poor quality healthcare services, and 3.6 million of them were due to non-utilization of healthcare services [5].

Access to healthcare services at early stage of disease onset could significantly reduce health complications, prevent transmission of communicable diseases, enhance successful treatment outcome, and reduce drug resistance [6–9]. Barriers to health and nutrition service is refers to the extent to which a population ‘gains access’ to financial, organizational and social or cultural barriers that limit the utilization of health and nutrition services [10]. However, considerable proportion of people has no access to healthcare services due to several sociocultural, financial or health facility related barriers [11–13]. Physical availability of infrastructure [11, 12, 14], distance to healthcare facility [15, 16], service cost [11, 17][, lack of education and information [12, 14], healthcare workers absenteeism [18], unavailability of services [11, 12], lack of transportation [17], long waiting time [14, 17] and economic hardship [19] are among several barriers of access to healthcare services. Patient satisfaction and quality of care are also one of the barriers of access to healthcare services [20, 21].

It is common that the occurrence of disease outbreak and other health risks are prevalent during the presence of natural disasters such as drought, flood, and earthquake [11, 22–24], due to essential health services delivery system disruption and environment related factors. The greater Horn of Africa is the region that has been highly affected by prolonged extreme drought and food insecurity, and millions are sustaining severe food insecurity in the region [25]. Uganda is one of the countries in greater Horn of Africa in which 19 districts in its north-east part are affected by drought and food insecurity [26]. Furthermore, previous study from Uganda confirms the existence of healthcare services access disparity across geographic areas and socioeconomic status [27]. The country is also experiencing different outbreaks of diseases such as Ebola and Yellow fever. The incidence rate of malaria is also increasing in the country-394 per 1000 population in 2022 in the drought affected districts (national routine data). Nine districts in Karamoja region are severely affected with drought and food insecurity, and 1.8 million people are currently faced acute food insecurity with 250, 000 people classified as an emergency level of food insecurity phase 4 in June to August 2022 [28]. Although there is no published evidence, information from the ground shows, drought and food insecurity in this region has significantly affected health and nutrition services delivery system. Health and nutrition services provision is also becoming a challenge in the region due to the staff-turnover and presence of social insecurity in the drought and food insecurity affected region to some extent.

Even though understanding barriers access to health and nutrition services is important in improving service delivery system, little information exist in drought and food insecurity affected areas. Furthermore, drought and food insecurity affected areas require sufficient access to healthcare services in order to overcome the impact of drought and food insecurity related public health complication. However, evidence on level of hardship to access healthcare services, and its barriers are lacking in drought and food insecurity affected areas. Thus, evidence on access to health and nutrition services is vital to support interventions that need to be implemented to reduce the public health consequences of drought and food insecurity in the region. Therefore, we aimed to explore the barriers of access to health and nutrition services in drought and food insecurity emergency affected districts in north-east Uganda.

## Methods

### Study design and area

We employed a descriptive qualitative method to explore barriers of access to health and nutrition services in Karamoja region and surrounding districts in north-east Uganda. Interview of this study were conducted from December 05 to 09, 2022. We conducted this study in 15 drought and food insecurity affected districts as a part of health and nutrition services assessment to improve response to drought and food insecurity emergency. The health and nutrition services assessment conducted was consisted availability of basic health and nutrition services, capacity of health facilities, service quality improvement actions and barriers to service access. The districts included to this study were Abim, Amudat, Kaabong, Karenga, Kotido, Moroto, Nabilatuk, Nakapiripirit, Napak, Omoro, Pader, Alebtong, Otuke, Kaberamaido, and Kapelebyong. Karamoja region and surrounding districts are the areas that severely affected by drought and food insecurity-classified as integrated food security phase classification (IPC) phase 2 and above food insecurity conditions [28]. Karamoja region is also known with its low socioeconomic status and pastoralist community. Thus, we conducted this study in the indicated study area to support the response being implemented to reduce the public health consequences of the drought and food insecurity.

### Sample size and sampling method

We considered all patients who sought health and nutrition services from health facilities and VHTs in the catchment area as study population. We selected subjects with maximum variation sampling method using sex and residence (rural versus urban) from all health system level (health center level I, II, III, IV and hospitals) in the districts. At least one subject and one VHT were interviewed per selected districts. The interview process was continued until information saturation reached. Information saturation was reached at 30 patients and 20 VHTs were interviewed.

### Inclusion and exclusion criteria

We interviewed adult subjects older than 18 years who sought health and nutrition services from the selected health facilities during the study period either from inpatient or outpatient department. The VHTs who work in the selected health facilities were also interviewed. However, severely sick subjects who need immediate medical attention, and who were mentally and physically incapable to be interviewed were excluded.

### Data collection tools and methods

We assessed the effect of drought and food insecurity on health and nutrition services access with one question. The question was “has it been harder for you to come and seek health and nutrition services from health facilities since the drought and food insecurity emergency occurred in the region? The response to this question was recorded with Likert’s scale having five levels such as “yes, very hard”, “yes, harder”, “yes, slightly harder”, “no, some as before” and “do not know”. To know the proportion of difficulty of accessing health and nutrition service, we categorized the response level of access to health and nutrition services into two categories: harder to access which consists “yes, very hard”, “yes, harder”, “yes, slightly harder”, and the same as before (the second category) during data analysis. Since none of the subject was responded “do not know”, we excluded it from the categories.

We collected data on barriers of access to health and nutrition services by researcher developed semi-structured interview guide which consists questions with probes. For example, the question asked was “what are the challenges of access to health and nutrition services since the drought and food insecurity emergency occurred in the region?”. The probes were also included lack of finance, lack of transportation, lack of services in health facility, no supplies (drug, laboratory reagents), absence of healthcare workers, poor healthcare workers communication, distance from the health facility. In-depth interview method was employed to collect data. The questionnaire was administered by trained health professionals who have experience on qualitative interview. The interview was conducted in pre-prepared suitable room in the selected health facilities. We recorded the qualitative data in tape recorder and field note.

### Data analysis

We used thematic data analysis approach by ATLAS.ti version 7.5.1.8 software after all responses were transcribed thoroughly. Thematic data analysis approach is important to examine the similarities of views of different participants to extract themes, categories and codes. After full understanding of the data by reading and rereading the data coding method was applied to find the codes emerged from the data. Data coding was conducted by dividing interviews into small number and the data analysis was discussed and the triangulation of the coding process conducted separately. After coding all interviews, the themes were searched from the similar codes and categories were created.

### Data quality assurance and trustworthiness

A two days training was given for data collectors on interview and probing method and ethical principles. The interview guide was evaluated by experienced professionals from ministry of health and World Health Organization (WHO) field team. Moreover, the data collection tool was piloted in five subjects in two health facilities. Based on the feedback from the professional and pilot study, the errors were corrected and clarity of the data collection improved. The study procedure including data analysis method were clearly presented, and thick description provided to assure trustworthiness of this study results.

## Results

**Fig. 1:**
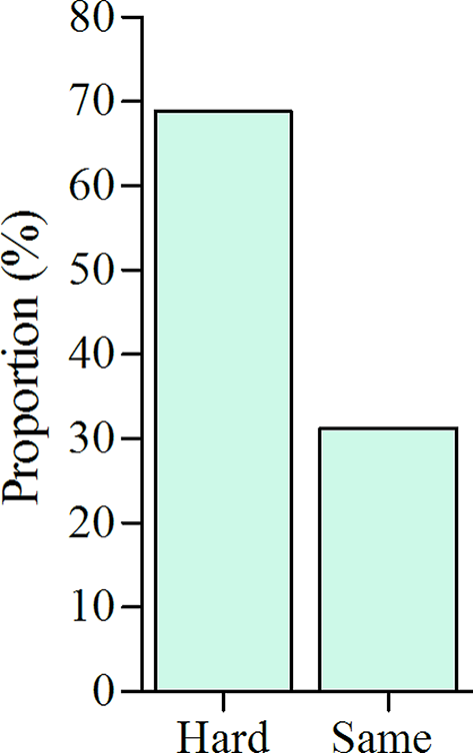
The effect of drought and food insecurity on health and nutrition services access from the perspective of subjects

Of the 30 interviewed subjects, 15 were female, while 17 residents of rural area, and the median age of the subjects was 29 years with interquartile range (IQR) of age 23 to 37 years. Almost half (46.8%) of the subjects visited outpatient department (OPD). Of the interviewed 20 VHTs, 11 were female and the median age of 31 years with the age range of 27 to 48 years. Most (68.8%) of the subjects reported that access to health and nutrition services was hard to them [Fig. 1].

The analysis of qualitative interview was generated four main themes which were reported by respondents as the barriers of access to health and nutrition services in drought and food insecurity affected districts [Table 1]. These themes included sociocultural and economic; environmental; health system, and subjects related barriers [Table 1].

**Table 1:** Barriers to access health and nutrition services in drought and food insecurity affected districts in Uganda, 2022

Codes		Themes	Categories
Fear of gender-based violence, mobile nature of the community, preference of traditional healers, and individual incongruent beliefs		Sociocultural and economic barriers	Sociocultural barriers
Poverty, high transportation cost, hunger, lack of crop product, lack of facemask to protect COVID-19 during health facility visit, lack of food, lack of money for referral and to buy medicine			Economic barriers
Drought, famine, absence of bridge to cross filled river during rainy season, bad road, bad weather condition		Environmental barriers	Seasonal related barriers
Lack of disarmament, domestic violence, fear of warriors, raiders and cattle stealing			Political barriers
Bad road, hard to reach areas, lack of nearby facility, poor communication network, long distance to health facility			Infrastructural barriers
Long waiting time, early closing of health facility, frequent drug stock out, lack of healthcare workers in the facility, absence of inpatients services, lack of ambulance, absence of required services, lack of privacy, lack of staff, poor sanitation, poor quality service, lack of supplies in health facility		Health system barriers	Health facility related barriers
Bad attitude of health workers, absenteeism of health workers, lack of experience, late coming for work, poor relationship with patient, stigma from healthcare workers			Health professional related barriers
Body weakness due to hunger, COVID-19 restriction, fear of COVID-19 and Ebola virus infection, frequent illness, medication side effect		Individual related barriers	Medical related barriers
Carelessness, alcohol consumption, fear to take medication, fear to explain their health problem, forgetfulness, ignorance, lack of time, job burden, knowledge gap, lack of information, lack of husband support			Patient related barriers

Table 2 depicts the frequency distribution of the four themes identified. Majority of the respondents were reported the four themes as the barriers of access to health and nutrition services under the drought and food insecurity emergency affected districts.

**Table 2:** Frequency distribution of the themes identified from qualitative analysis

Theme		n (%)
Theme one: sociocultural and economic barriers to access health and nutrition services	Yes	44(88)
	No	6(12)
Theme two: Environmental barriers to access health and nutrition services	Yes	35(70)
	No	15(30)
Theme three: Health system-related barriers to access health and nutrition services	Yes	43(86.0)
	No	7(14)
Theme four: Individual patient-related barriers to access health and nutrition services	Yes	42(84)
	No	8(16)

### Theme one: sociocultural and economic barriers to access health and nutrition services

#### Sociocultural barriers

Most of the respondents raised the effect of cultural malpractice, fear of gender-based violence (GBV), mobile nature of the community, preference of traditional healers, and individual wrong beliefs as the main barriers that limit access to health and nutrition services [Tables 1 and 2]. A VHT worker explained the effect of GBV on access to health and nutrition services as, *“There are various gender-based violence in many households which hinder families to take children to health facilities on time (35 years old, female VHT worker).”* A subject who was interviewed also explained the problem of mobile nature of the community, stating as, “*Distance from my home to health facility is very long because we have moved from our original place to find pasture for our cattle (A 42 years old, male subject).”*

#### Economic barriers

Respondents reported that poverty or lack of money, expensive transportation cost, and lack of food to eat, lack of facemasks to protect COVID-19 as the main barriers of health and nutrition service access. A 40 years old female subject explained the effect of lack of money and food on service access as follows: *“There is no money to pay for transportation and food to eat. Sometimes, when you are hungry and your body is weak to walk to health facility, stay sick at home is better.”* Another respondent also said, *“Due to the drought, our cattle has died, therefore no money to buy drugs and food, even some drugs require food to swallow (A 30 years old, male subject).”*

### Theme two: environmental barriers to access health and nutrition services

#### Seasonal related barriers

According to the respondents, drought, hunger, flood due to sudden over flow of river (in some districts), roadblocks, and bad weather were seasonal related barriers of access to health and nutrition services. One subject explained the effect of environment on access to health and nutrition services as follows, *“The main reason for not use health facility is due to flooded roads and lack of money to use boat to cross the flooded road (A 36 years old, female subject).”* Another subject interviewed said, *“…the other factors that hinder me to use health and nutrition services are poor accessible road and high cost of transportation during the rainy season (A 23 years old, female participants).”*

#### Political barriers

The respondents raised different political barriers under environmental theme which hindered them from access to health and nutrition services. These were lack of disarmament, domestic violence, fear of the warriors and raiders, insecurity, and fear of cattle stealing. One VHT worker, who was interviewed, explained these situations as follows, *“In my opinion, the main factor that prevent them (the subjects) to use health and nutrition service is insecurity, it makes them relocate to different place (A 37 years old female VHT worker).”* Another subject also added, *“…due to disarmament we have no weapon to defend ourselves from warriors who attack us in the way to health facility, sometimes even within health facility (A 50 years old male subject).”*

#### Infrastructural related barriers

Infrastructure-related barrier was one of the categories that emerged under the environmental related theme. The majority of the participants reported that bad roads, hard-to-reach areas, poor communication network, and long distance to health facility were the main barriers that hinder the patients from accessing health and nutrition services. One subject interviewed described as below: *“In the event of an emergency, the poor road network complicates the use of ambulance for referral service. Poor roads and long distances make harder to reach to the facility (A 25years old male subject).”* Another subject interviewed said, “*Distance to health facility is the main challenge to get to health facility to access service. As Boda boda (motor bicycle) is a common transportation means in our area, it is not suitable for the subject especially on bad road. Poor network connectivity is also the main challenge to get help from health facility. The road is not good to go to bigger hospital, and there is no ambulance during bad condition (A 25years old male subject).”*

### Theme three: health system-related barriers to access health and nutrition services

#### Health facility-related barriers

Health facility related barrier was one of the two categories that emerged under health system related barriers theme. Study participants mentioned that waiting time, early closure of the health facility, frequent drug stock-outs, lack of health workers, lack of inpatient services, lack of nutritional services, lack of privacy, poor sanitation in health facility, and poor quality of service were the barriers of access to health and nutrition services. A subject explained as, “*How I go to health facility because very few health workers present in the health facility to deliver service on some days which leads to very long waiting hours, and many subjects to wait for the service (A 21years old female subject).”*Another subject was also explained *“there is no medicines in the health center, few services and health professionals are present in the health center (A 31years old female subject).”*

#### Health professional-related barriers

Respondents raised several health professional related barriers that affect access to health and nutrition services. Bad attitudes toward health workers, absence of health workers in the work place, poor healthcare workers experience, and lateness to come to work, poor relationships between healthcare workers and patients, and stigma were the most common health professional related barriers of access to health and nutrition services. A subject interviewed said, “*Some healthcare workers are impolite and use unethical bad language and words when treat the subject (35years old, female subject).”* Another subject also explained as, “*Health workers do not work beyond 2 PM, and they chase you to go back, if you come in the afternoon (A 37 years old female subject).”*

### Theme four: individual subject-related barriers to access health and nutrition services

#### Medical barriers

Most of the respondents raised barriers that related to medical situation under individual patient related theme. Body weakness, COVID-19 related restriction, fear of COVID-19 and Ebola virus infection, repeatedly sickness, and medication side effects were reported as the barriers that related to medical conditions. A VHT worker said, “*Some people fear contracting of corona and Ebola from health facilities (A 38 years old female VHT worker)”* Moreover, the same VHT worker explained, “*… children are fall sick frequently, but bringing them to a facility is hard to the family because they fear contracting either corona or Ebola (38 years old female VHT worker).”* A subject was also explained, “*…sometimes when your body is weak, you cannot walk to the health facility, rather you prefer staying at home with sickness (A 37 years old male subject).”*

#### Subject-related barriers

Subject-related barrier was one of the categories that emerged under individual-related barriers theme. The respondents raised different reasons that prevent them to access health and nutrition services in the study area. Carelessness, fear of taking medication, fear of explaining about illness, forgetfulness to go to hospital, ignorance, lack of time, job burden, knowledge gap and lack of information, and lack of support from spouse were the most common subject related barriers to use health and nutrition service. A VHT worker mentioned the effect of subject related barriers as, *“Most of the community members prefer working in garden instead of coming to health facility to get support for their illness. In addition, some people fear taking medicine in empty stomach because getting food is very hard for them (A 28 years old female VHT worker).”* A subject explained the impact of lack of knowledge to access health and nutrition services as, *“We do not come to health facility because we do not know the importance of health and nutrition care (A 50 years old female subject).”*

## Discussion

About three-fourth (68.8%) of subjects reported that access to health and nutrition services were hard to them since drought and food insecurity emergency happen. The current study identified four themes, nine categories and several codes as the barriers of access to health and nutrition services in drought and food insecurity affected districts in north-east Uganda. The identified themes include sociocultural and economic; environmental; health system, and subjects related barriers.

In the current study considerable number of subjects reported as access to health and nutrition services were hard to them. Although we couldn’t find similar previous study that reported the hardship level of the subjects to access health and nutrition services under drought and food insecurity emergency, available evidence confirms that 52% of African population have no access to health services [3]. Moreover, half of the world population have no access to essential health services [2]. This evidence confirms the finding of the present study in which considerable proportion of subjects were reported the hardship they face to access health and nutrition services under drought and food insecurity situation. This hardship to access health and nutrition services might be exacerbated by prolonged drought in the area, which requires intensive interventions to reduce its public health consequences.

Based on the previous studies conducted, sociocultural barrier is one of the important barriers of access to health services [12, 29, 30]. These studies’ findings were consistent with the current study finding in which sociocultural barriers such as mobile nature of the community, preference of traditional healers and individual beliefs were reported as the barriers of access to health and nutrition services. Most of the time, pastoralist community travels long distance away from their original place where health facilities are inaccessible to find grass land and water for their cattle. This mobile nature of the community could limit access to health services [29, 30]. Although mobile model of health and nutrition services delivery system is recommended and practicing in different areas to overcome barrier related to mobile nature of pastoralist community recently [29, 30], still it imposes obstacle to access to health and nutrition services.

In the present study, respondents reported that some patients prefer traditional healers than modern medicine that is being provided by health facility. This finding is supported by a previous study result in which 52% of the patients reported traditional healers as their first choice of treatment than conventional treatment [31]. This could be due to accessibility and price of service of traditional healers in the study area.

Individual patient belief and perception were also reported as the barrier of access to health and nutrition services in the present study. This finding is consistent with the previous study reported by Alwan et al. [32] in which individual belief was a barrier of access to healthcare services. A previous review study was also reported [20] similar finding with the current study in which individual belief was the barrier of access to healthcare and nutrition services.

Economic barrier was commonly reported by the subjes in the present study. Previous studies confirm that poverty is the main barrier of access to healthcare services in different settings [19, 33]. Moreover, poor people have low access to healthcare service in LMICs [33], which is consistent with the present study finding. Furthermore, in present study, subjects explained high transportation cost, hunger, lack of food, and money as the economic barriers of access to health and nutrition services. A previous study finding [14] is similar with the present study result in which economic hardship was a major barrier of access to healthcare and nutrition services. In addition, the previous studies showed that cost of healthcare seeking is a barrier of access to health services [20, 34] which is similar with the present study finding in which high transportation cost is the barrier of access to health and nutrition services.

Seasonal related barriers were commonly reported as the barriers of access to health and nutrition services in the current study. Prolonged drought is undergoing in the horn of Africa which is also significantly affected the community resided in the present study area [25]. The persistent drought in the area leads to extreme food insecurity which could in turn to famine and lack of money to access health and nutrition services. Although we could not find similar study on the effect of drought on health and nutrition service access, previous evidence indicates that drought could affect the capacity of health system in routine health services deliver [35]. Wet season could also reduce access to healthcare services [36]. This result is consistent with the present study finding in which rainy season was reported as the barriers of access to health and nutrition services due to flood and road erosion which leads to high transportation cost. Lack of road during the rainy season and flooding in some areas were also explained as seasonal barriers of access to health and nutrition services. A previous study showed that transport connectivity to health facilities is a significant determinant of access to healthcare [37, 38] which is similar with the present study finding in which poor road was reported as the barrier to access health and nutrition services.

In the present study infrastructures such as poor road, long distance to healthcare facility, living in hard-to-reach areas, and poor communication network were reported by the subjects as the barriers of access to health and nutrition services. Previous studies’ results [34, 37] are consistent with the present study finding in which poor road was the barrier of access to health and nutrition services. Moreover, one study found that long distance to health facility appeared to be a barrier of access to healthcare services [39], which was in line with the current study finding in which long distance to health facility was a barrier of access to health and nutrition services. Majority of community in the study area are pastoralist and living in hard-to-reach areas which was identified as a barrier of health and nutrition services in the current study area.

Poor communication network was also reported as the barrier of access to health and nutrition services in the present study. Although we could not find similar study that reported on the effect of communication network on health and nutrition services access, the importance of communication network for health services provision is well documented [40]. The present study finding confirmed poor communication network in the study area was a barrier of access to health and nutrition services.

Weapon disarmament, fear of warriors and raiders, and fear of cattle stealing were political environment related barriers of access to health and nutrition services that frequently reported by the respondents in the present study. A study on insecurity to health service delivery reported from Democratic Republic of Congo (DRC) found that insecurity affects access to health services and quality through violence, mobility restriction and resources unavailability [41]. This finding supports the current study finding in which insecurity due to warriors and raiders in the study area was reported as the main barrier of access to health and nutrition services.

Long waiting time, early closing of health facility, drug stock out, lack of healthcare workers in the facility, lack of ambulance, lack of privacy, poor sanitation in health facility and poor-quality services were health facility related barriers that identified in the present study. Not being able to get a service when needed can affect health seeking behavior of the patients and it becomes the barrier for access to health services. Studies conducted in Australia [11, 14] found that long waiting time appeared to be a contributing factor of access to general practitioner [11, 14]. This finding is consistent with the current study result in which long waiting time to obtain health and nutrition services (the commonly reported barrier). Moreover, a previous study reported that emergency room waiting time was the barrier of access to health services among Syrian refugee in United State [32].

Previous studies identified that patient-healthcare workers relation was the barrier of access to health services [32, 42–44]. These studies’ results were similar with the current study finding in which healthcare workers related factors were the barriers of access to health and nutrition services. Moreover, poor patient-provider communication was reported as a barrier of access to health services [44]. This report is consistent with the present study finding in which unethical communication of healthcare workers with patient was the barrier of access to health and nutrition services.

Previous studies confirm that COVID-19 related restriction and fear are the barriers of access to health services [45, 46]. These findings are similar with the present study results in which COVID-19 and Ebola related restriction and fear were barrier to access health and nutrition services.

The previous studies revealed that individual subject related factors are associated with access to healthcare services [16, 42]. These studies results are consistent with the present study finding in which several subject related factors reported as the barriers of health and nutrition services.

The findings of the current study have clearly identified the modifiable barriers of access to health and nutrition services in drought and food insecurity affected area. Therefore, short- and long-term interventions implemented in the area should take in consideration the identified barriers of access to health and nutrition services in the area.

The main limitation of this study is that the qualitative nature of the study design, which could miss some important quantitative information that limits the comprehensiveness of the current study findings. Moreover, data on important sociodemographic variables such as education status and economic status were not collected. These limitations couldn’t enable us to further explore the barriers of access to health and nutrition services through triangulating quantitative and qualitative data. Thus, the perceived barriers of access to health and nutrition services may not be limited to the factors presented in the present study. Future study should employ a mixed method to capture important quantitative and qualitative data on the barriers of access to health and nutrition services in drought and food insecurity affected setting.

## Conclusion

This study identified several modifiable perceived barriers of access to health and nutrition services in drought and food insecurity affected districts in Uganda. A comprehensive intervention that aimed at addressing the identified sociocultural, economic, environmental, health system and individual subject related perceived barriers of access to health and nutrition services are required to effectively improve access to health and nutrition services.

## Data Availability

All data used in this study are presented in the manuscript. However, the data is available in the hand of corresponding author and can be accessed on the reasonable request.

## Abbreviations

COVID: 19-Coronavirus disease 2019
GBV: gender based violence
IPC: Integrated food security phase classification
IQR: Interquartile range
LMICs: Low-and middle-income countries
OPD: Outpatient department
SDGs: Sustainable development goals
VHTs: Village health teams
WHO: World Health Organization
